# RGS proteins and their roles in cancer: friend or foe?

**DOI:** 10.1186/s12935-023-02932-8

**Published:** 2023-04-28

**Authors:** Lin Li, Qiang Xu, Chao Tang

**Affiliations:** 1 National Clinical Research Center for Child Health of the Children’s Hospital, Zhejiang University School of Medicine, No. 3333, Binsheng Rd., Hangzhou, 310052 People’s Republic of China; 2 Department of Urology, Third Affiliated Hospital of the Second Military Medical University, Shanghai, 201805 China

**Keywords:** RGS, GPCR, Epigenetic regulation, Transcription, Protein expression, Cancer

## Abstract

As negative modulators of G-protein-coupled receptors (GPCRs) signaling, regulators of G protein signaling (RGS) proteins facilitate various downstream cellular signalings through regulating kinds of heterotrimeric G proteins by stimulating the guanosine triphosphatase (GTPase) activity of G-protein α (Gα) subunits. The expression of RGS proteins is dynamically and precisely mediated by several different mechanisms including epigenetic regulation, transcriptional regulation -and post-translational regulation. Emerging evidence has shown that RGS proteins act as important mediators in controlling essential cellular processes including cell proliferation, survival -and death via regulating downstream cellular signaling activities, indicating that RGS proteins are fundamentally involved in sustaining normal physiological functions and dysregulation of RGS proteins (such as aberrant expression of RGS proteins) is closely associated with pathologies of many diseases such as cancer. In this review, we summarize the molecular mechanisms governing the expression of RGS proteins, and further discuss the relationship of RGS proteins and cancer.

## Introduction

Regulator of G protein signaling (RGS) proteins, which modulate G protein-coupled receptors (GPCRs, located in cellular membrane and transmit outer signals into the intra-cellular environment) function, facilitate various downstream cellular signaling through regulating kinds of heterotrimeric G proteins by the acceleration of the intrinsic guanosine triphosphatase (GTPase) activity of their Gα subunits [1, 2]. In most cases, regulation of RGS proteins gives rise to the inhibition of multiple downstream G protein signaling pathways [3], and RGS proteins are thereby recognized by many researchers as the important downstream nodes of those GPCRs [4]. Since the discovery of RGS proteins in different species including yeast, *Caenorhabditis elegans* as well as mammalian cells in the 1990s [5–8], their pivotal role in altering cell proliferation, survival and death via controlling downstream cellular signaling activities has provided with the evidence that RGS proteins are potentially involved in sustaining normal physiological functions and that dysregulation of RGS proteins is closely associated with pathologies of many diseases such as cancer. In this review, we summarize the history and structure of RGS, and its role in cancer, and further discuss the molecular mechanisms governing the expression of RGS proteins, offering implications of these new discoveries for novel targeted drug development and related cancer therapy in the future.

### A brief description for history of RGS protein discovery

**Fig. 1:**
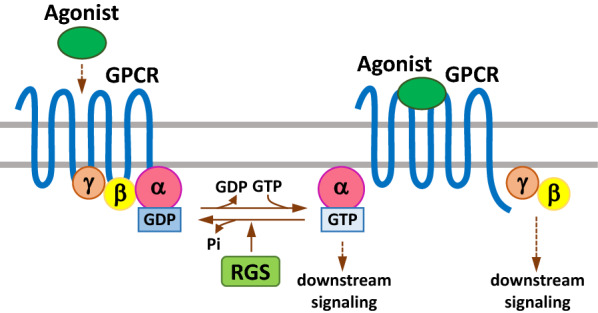
The schematic graph showing canonical regulation of GPCR signaling by RGS proteins. Upon bound with some agonist, GPCRs undergo a conformation change that facilitates the exchange of GDP for GTP on the a subunit of the heterotrimeric complex. Both GTP-bound Ga in the active form and the released Gbg dimer can subsequently stimulate the corresponding downstream signaling. RGS proteins are GAPs for Ga, which function to terminate signalling through GPCRs by accelerating the intrinsic GTPase activity of Ga and promoting reassociation of the heterotrimeric complex with the receptor at the cell membrane

The discovery of RGS proteins is achieved through a series of studies by different experimental systems. In the period of 1995 to 1997, experiments in *Saccharomyces cerevisiae* revealed the novel factor Sst2 that modulates Gpa1 (a G subunit in yeast) is involved in the regulation of pheromone sensitivity [9, 10]. Additional work performed in 1996 using the nematode *C. elegans* detected the mutations in the gene egl-10, which reflected mutations in GOA1 that participates in other signalings and in mammals are analogous to G proteins [7]. Later in 1997, egl-10 and Sst2 were genes found to share similar sequences to each other, and then several groups proposed that they could be a potentially new class of GPCR regulators in mammals [1, 7, 8]. Subsequently, the importance of these novel findings were proved by various experiments in rapid publication of papers from several independent research groups, and the main results and conclusions are as follows (Fig. 1): (1) RGS proteins bound with the ∝ subunits of G-protein directly; (2) the interaction of RGS with these subunits potentiated the GTP hydrolysis rate by G∝ (referring to GAP activity); (3) different RGS proteins specifically recognized their targeted G subunits, respectively [2, 6, 11]. The mechanisms underlying RGS protein activity regulation was further deciphered by a report later, showing how those proteins catalytically promoted GTP hydrolysis by G∝ subunits through stabilizing the transition state for GTP hydrolysis, and this finding established the canonical functions of RGS proteins, including GTPase-activating or GAP activity [5].

### RGS protein family and RGS protein structure

**Fig. 2:**
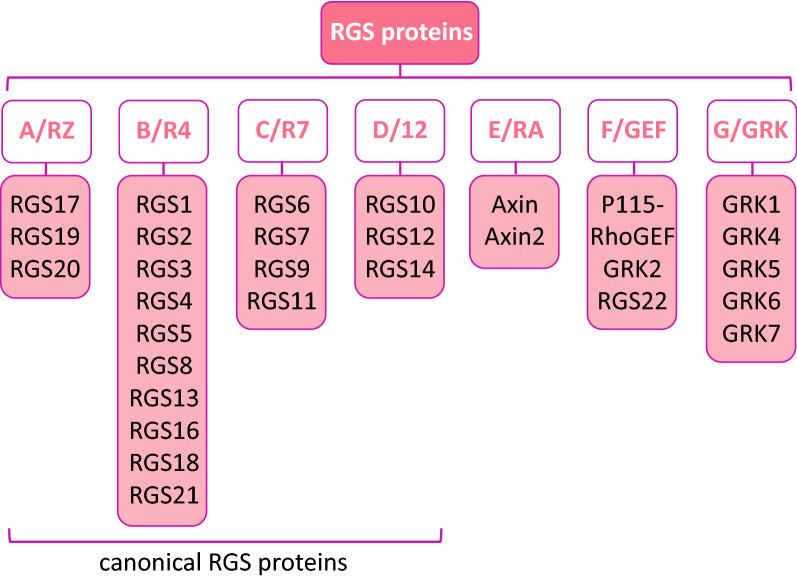
The schematic lists of the family of RGS proteins. The different RGS proteins were established and were named after their prototypical members, and the A/RZ, B/R4, C/R7 and D/R12 families constitute the canonical RGS proteins

In mammals, members of the R4 family of RGS proteins were the first ones clarified and studied, which are now typified by RGS4. Among all the RGS proteins, the RGS4 family represents the least structurally and functionally complex. To date, members of RGS proteins have been divided into different families, based on their varied structures and functions. The different RGS proteins were established and were named after their prototypical members, including A/RZ family, B/R4 family, C/R7 family, D/R12 family, E/RA family, F/GEF family and G/GRK family, among which, the A/RZ, B/R4, C/R7 and D/R12 families constitute the canonical RGS proteins [12] (Fig. 2). All the canonical RGS proteins possess the conserved RGS domain with a length of approximately 120 amino acids (aa), which is consisted of nine α-helices structures that can be subdivided further into two subdomains [4]: (1) the first subdomain that forms a smaller helix bundle and is consisted of series of helices including αI, II, III, VIII and IX; and (2) the larger bundle subdomain comprises four helices including αIV, V, VI and VII [13]. Different from the B/R4 family, which is with RGS4 as its prototypical member, the other RGS proteins contain multiple domains that participate in the interaction with various proteins beyond the G∝ and possess more complex domains of cellular function, such as the domain from presence in proteins PSD-95, Dlg and ZO-1/2 domains (PDZ domains), G-protein ∝-like domains (GGL), domain present in disheveled and axin (DAX) domains, kinase domains, Dbl homology/pleckstrin homology domains (DH), G-protein regulatory motif (GoLoco) domains, ∝-catenin–binding domains as well as Ras-binding domains (RBD). To date, there have been at least 20 distinct RGS proteins classified, which play various regulatory roles and can be divided into seven families [14]: A/RZ family includes RGS17, RGS19 and RGS20; B/R4 family includes RGS1, RGS2, RGS3, RGS4, RGS5, RGS8, RGS13, RGS16, RGS18, RGS21; C/R7 family includes RGS6, RGS7, RGS9 and RGS11; D/R12 family includes RGS10, RGS12 and RGS14; E/RA family includes Axin and Axin2; F/GEF family includes P115-RhoGEF, GRK2 and RGS22; G/GRK family includes GRK1, GRK4, GRK5, GRK6 and GRK7. However, as some RGS proteins with a number of genetic variations continue to be revealed, the number of new RGS proteins discovered is still increasing, such as the RGS6 protein that possesses several splicing variants with varied functions and cellular localization [15], and the RGS14 protein with genetic variants that disrupt downstream signaling activation [16].

A previous study in 1997 has already identified the structure of RGS protein with the classification of RH (RGS homology) domain [13]. In that study, the crucial structural determinants of the interaction of RGS protein with G determinants of the interaction of RGS protein with G∝ have also been revealed, which has established the structural basis for its GAP activity. As their function of negative mediators in G-protein signaling, RGS proteins are found to mainly exert their effects on regulating GAP activity on α subunits of G-proteins, particularly the Gi/o and Gq families of G-proteins. Although there are yet no reports confirming the GAP activity of any RGS domain against Gαs, emerging evidence has come out that RGS proteins are able to indirectly regulate Gαs downstream signaling through their interaction with subtypes of adenylate cyclase (AC) [17]. Despite the functions of the non-RH domains in RGS proteins, the RH domain is still most studied today, which is attracting the attention of researchers around the world for identifying and developing novel inhibitors to suppress RGS activity to control kinds of downstream cellular signaling and to further help provide interventions of related diseases.

### Mechanisms regulating RGS expression

**Fig. 3:**
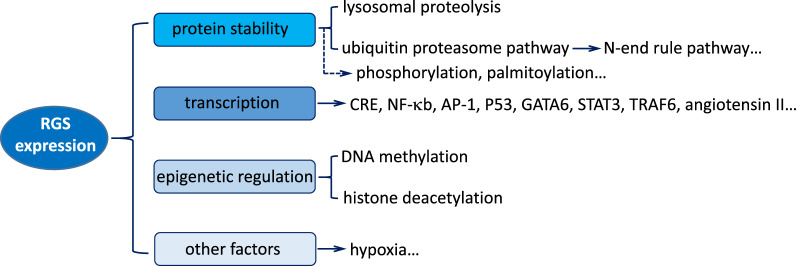
Summary of mechanisms regulating RGS proteins expression. RGS protein expression is affected by different factors, including regulation of protein stability (degradation regulation and post-translational modification), transcriptional regulation, epigenetic regulation (DNA methylation and histone deacetylation), and other factors such as hypoxia

Previous studies have provided evidence that the levels of RGS proteins are initially associated with the mechanisms that mediate the local concentration of those proteins at the site of a cell signaling. In addition, RGSexpression is also affected by other factors, including its regulation of protein stability, regulation at transcriptional levels, epigenetic regulation, regulation of subcellular localization as well as the environmental conditions such as hypoxia [3, 18–25] (Fig. 3), which allow RGS protein levels to be altered at both an acute and a chronic manner.

### Regulation of RGS protein stability

Protein degradation is a dynamic but essential process employed by all of the cells to efficiently and precisely modulate the levels of stable proteins, resulting in the proper functions of those proteins for cells [26, 27]. The degradation commonly undergoes through either of the two ways, including (1) lysosomal proteolysis pathway; and (2) ubiquitin proteasome pathway [28]. Among the two pathways, the lysosomal proteolysis is triggered by proteins such as the lysosomal engulf proteins and the associated digestive enzymes, while the ubiquitin proteasome pathway degrades proteins through regulating poly-ubiquitination of the targeted proteins. During this process, the proteins that complete poly-ubiquitination can be recognized by a large and complex molecular machine, the proteasome complex, which subsequently binds to and degrades the targeted proteins eventually. Evidence has emerged that multiple enzymes participate in regulating the ubiquitin proteasome-dependent protein degradation, including the ubiquitin-activating enzymes (E1), the ubiquitin-conjugating enzymes (E2) and the ubiquitin ligases (E3), and compared to the lysosomal degradation pathway, ubiquitin proteasome pathway requires more energy.

The expression of RGS proteins is affected by their protein stability. In the past decade, previous studies have demonstrated the potential role of RGS4 as a target for degradation by proteasome [29]. The mechanisms underlying RGS4 protein degradation is due the regulation by the N-end rule pathway, a subset of the ubiquitin-mediated proteolytic pathway. The N-end rule pathway potentiates the targeted protein degradation by its recognition of the certain amino acid residues at the N-termini of those proteins. Based on the N-termini residue, the N-end rule pathway can be further subdivided into three types in eukaryotes, including the Arg/N-end rule pathway, the Ac/N-end rule pathway and the Pro/N-end rule pathway, which correspondingly recognizes the basic, acidic, amidated, and bulky hydrophobic N-termini residues, the N^α^-terminally acetylated N-termini residues, and the N-termini-Pro residue or a Pro residue at position 2 in the presence of adjoining sequence motifs [30, 31]. A previous report has indicated that blocking of the N-end rule pathway efficiently suppresses the degradation and ubiquitination of RGS4 proteins in the reticulocyte lysate system [29], while the existence of MG132, a kind of proteasome inhibitor, inhibits RGS4 protein degradation but concurrently increased the protein levels of RGS4 that were poly-ubiquitinated, which further provides with the evidence that RGS4 protein is degraded through the N-end rule pathway. Interestingly, RGS4 protein degradation is also observed to be regulated by nitric oxide, which oxidizes the N-termini-cysteine residue that is necessary for the subsequent arginylation [32]. In addition to RGS4 protein, some other RGS proteins are also involved in the regulation by the N-end rule pathway, including RGS16, RGS5 as well as RGS2 [32–34]. Particularly, unlike RGS4, the proteasomal degradation of RGS2 protein requires a protein complex that includes DNA damage binding protein 1 (DDB1), F-box only protein 44 (FBXO44) and cullin 4B (CUL4B) [35]. Recently, the expression of RGS proteins has also been found to be affected by their stability through other mechanisms, such as the RGS9-2 protein that is specifically expressed in striatal neurons and functions mainly in the brain [36]. It is reported that, different from many other RGS proteins, RGS9-2 protein is mainly mediated by the lysosomal degradation pathway, and the proteolytic stability of RGS9-2 protein is controlled by R7 family binding protein (R7BP), which is determined as a newly discovered partner for RGS9-2. Another example is the RGS7 protein that belongs to the R7 family of RGS proteins, whose stabilization is specifically modulated by the binding partner Gβ5 [37]. Post-translational modifications, such as protein phosphorylation, are also confirmed to be strongly associated with RGS protein stabilization. Phosphorylation plays a key role in regulating the activities of a variety of cellular signaling pathways by affecting the expression, localization and stabilization of the targeted proteins, which consequently leads to alterations in cell proliferation, apoptosis, survival, mobility and possible malignancy as well [38]. To date, multiple phosphorylating sites have been figured out in different RGS proteins, which give rise to alterations in stability and activity of the RGS proteins. One example is the RGS16 protein that is phosphorylated constitutively at serine 194 (Ser 194) site and undergoes dynamical phosphorylation at the Ser 53 site induced by the activation of α2A-adrenoceptor. The altered phosphorylation levels of RGS16 protein contribute to the inhibition of GAP activity in RGS16, whereas RG16 phosphorylation at Tyr 168 potentiates not only the GAP activity but also the stability of RGS16 protein [39, 40], suggesting RGS16 levels and functions are closely associated with its phosphorylation status. One phenomenon that attracts many researchers’ attention is that, during the phosphorylation regulation, different RGS proteins can be affected by one same kinase. One famous example is the protein kinase A (PKA), a cAMP-dependent protein kinase that is involved in affecting various diseases including cancers in human [41, 42]. PKA is demonstrated to enhance RGS13 activity to negatively regulate CREB-induced transcription of target genes by facilitating the nuclear localization of RGS13, and concurrently, PKA blocks the proteasome degradation of RGS13 protein by its induction in phosphorylation at Thr 41 of RGS13 [43]. Similarly, PKA activation is also involved in promoting the nuclear trans-location of RGS10 protein. In addition to phosphorylation, other post-translational modifications have also been shown to be associated with RGS protein stability, such as protein palmitoylation [44, 45].

### Regulation of RGS transcription

The expression of RGS proteins is additionally observed to be affected by alterations in RGS transcription. Owing to the great work in the past decade, various binding sites for different transcription factors have been identified within the promoter regions of kinds of *RGS* genes, suggesting the potentially direct regulation of RGS transcription by the multiple transcription factors. A highly conserved sequence of cAMP response element (CRE) binding site was initially isolated and characterized in the promoter of mouse *Rgs2* gene, and mutations in the CRE site down-regulate the activity of *RGS2* gene promoter, indicating the fundamental role of this CRE site for *RGS2* transcription [46]. In addition to RGS2, the CRE site was also identified later in the promoter regions of genes *RGS4* and *RGS5* [47, 48], providing with the evidence that this CRE site is required for the transcriptional process of multiple *RGS* genes. Despite the common CRE site locating at the promoters of different *RGS* genes, the effect on *RGS* gene transcription is particularly dependent on the transcription factor that binds to the CRE site. For example, association of transcription factor CRE-binding protein (CREB) to the CRE site in the promoter region of *RGS2* gene trans-activates *RGS2* gene, whereas binding of CRE-modulator (CREM, a related factor of CREB) to the promoter of *RGS5* gene conversely restrains *RGS5* transcription, unexpectedly. One hypothesis is that those two factors, CREB and CREM, might possess competition effects and thereby counteract each other at the CRE site at the promoters in different *RGS* genes, resulting in the dynamic regulation of RGS expression. Recently, other sites have also been characterized in the promoter of different *RGS* genes, including the NF-κB binding site, the AP-1 binding site as well as the P53 binding site [49–51], which not only control the transcription of different *RGS* genes but also synergistically facilitate the transcription in one *RGS* gene. For example, in colonic muscle cells, the IL-1 β -induced RGS4 transcription is co-mediated by different transcriptional factors, among which, GATA-6 and NF-κB up-regulate RGS4 transcription in response to IL-1β stimulation, whereas AP-1 down-regulates the transcription of *RGS4* gene [50, 52, 53]. Similar phenomena can be also observed in other *RGS* genes, such as RGS2 that is regulated by transcription factors including CRTEB and STAT3, and RGS16 that is modulated by both P53 and NF-κB [54–56]. It is likely that these various regulations contribute to the more complex expression pattern, which may cause a quick and suitable response to the extracellular stimulation. As discussed above, one *RGS* gene can be concurrently modulated by multiple transcription factors, while on the other hand, one transcription factor is able to mediate the transcription of different *RGS* genes. Some examples can be found from the previous publications, such as NF-κB that trans-activates both *RGS4* and *RGS16* [52, 54], and STAT that trans-activates both *RGS2* and *RGS7* [57, 58]. Intriguingly, some transcription factors exert the opposite effects on regulating the transcription in different *RGS* genes. One example is P53, which potentiates the transcription of *RGS16* gene in human EB1 colon cancer cells [56] but inhibits the transcription of *RGS13* gene in mast cells and B lymphocytes [51], indicating that the regulation of one transcription factor in different *RGS* genes is differently dependent on the specific cell context. Given that transcriptional regulators usually function as multi-protein complexes to cooperatively modulate target gene expression and the expression of each component in these multi-protein complexes may vary in certain tissues and cell types, it is thereby likely that there is significant variation in the effect of a specific transcription factor on RGS expression in different tissues. Thus, extensive investigation and characterization is required to fully understand the regulation pattern of RGS expression, which may help provide opportunities for more promising therapeutic approaches to control RGS expression in clinic.

Recently, some other factors have also been found to regulate *RGS* transcription, and one example is tumor necrosis factor receptor-associated factor 6 (TRAF6), which negatively mediates EMT in choriocarcinoma cells by repressing *RGS2* transcription [59]. In addition, angiotensin II is shown to regulate *RGS2* mRNA expression in vascular smooth muscle cells cultured in vitro [60]. Recently, micro-RNAs (miR), a group of evolutionarily conserved small regulatory RNAs that participate in the regulation of diverse fundamental biological processes [61], are found to directly target some *RGS* mRNAs and affect their levels, such as RGS17 that is proven to be the direct target of miR-203 [62], RGS12 that is targeted by miR-204-5p in stress-induced pathology [63], RGS4 that is regulated by miR-107 in hepatocellular carcinoma [64], and RGS3 that is mediated by miR-133a in gastric cancer [65].

Emerging evidence has also shown the bi-directional regulations between multiple transcription factors and different RGS proteins. Some RGS proteins have been found to bind with potential transcription factors directly and thereby facilitate their functions, such as RGS2 that directly interacts with STAT3 and thus suppresses STAT3-induced transcription activation [43], and RGS13 that suppresses CREB-induced transcription through its translocation into the nucleus where it forms a protein complex with the transcription factors CREB as well as CBP/P300 [66]. Given that *RGS* gene transcription is modulated by both transcription factors STAT3 and CREB, it is likely that RGS protein may be involved in the mutual transcriptional regulation through feedback mechanisms.

### Epigenetic regulation and RGS expression

Epigenetic modifications control gene expression by altering the structure of nuclear chromatin, particularly including the regulation of histone structures, which is intimately connected to both human development and disease pathogenesis [67]. The epigenetic modifications can occur in both DNA and histones (two classical examples of epigenetic modifications) and consequently activate or suppress target gene expression through the mediation of accessibility of potential transcription factors via tightening or loosening the chromatin complex [68]. As a representative for epigenetic modification in DNA, DNA methylation at cytosine is controlled by a kind of enzyme named the DNA methyltransferase enzymes (DNMTs), and this methylation regulation gives rise to inhibition of target gene expression [69]. Unlike the epigenetic modification in DNA, histones undergo acetylation to regulate target gene expression, during which the enzymes histone acetyltransferase (HATs) that acetylate histones cause gene expression activation, whereas the histone deacetylases (HDACs) that deacetylate histones exert negative effects on gene expression [70]. Previous studies have reported that the *RGS* gene expression is associated with the DNA methylation status and histone deacetylation status in *RGS* genes. An earlier publication on the relationship between RGS expression and *RGS* gene epigenetic modification indicated that the methylation status of *RGS16* gene promoter was significantly increased in breast cancer cells, where the RGS16 protein expression was obviously reduced [71]. Likewise, the alterations in methylation status of *RGS2* gene promoter were later discovered in prostate cancer cells, where the protein expression of RGS2 was inversely correlated with the methylation status of *RGS2* gene promoter, while suppression of *RGS2* DNA methylation effectively attenuated the protein expression of RGS2 in those cells, proving RGS2 expression is precisely controlled by the mediation in its gene promoter [72]. In addition, another example can be explained in bladder cancer cells, where the multifunctional protein Ubiquitin-like with PHD and ring-finger domain 1 (UHRF1)-enhanced methylation status in *RGS2* gene promoter repressed RGS2 expression but promoted cancer progression [73]. Similarly, a DNMT enzymes-induced increase in methylation status of *RGS10* gene promoter was found in ovarian cancer, particularly in the ovarian cancer cells with chemoresistance, which was inversely correlated with RGS10 expression in those chemoresistant ovarian cancer cells, compared with the cells of chemosensitive counterparts [74, 75]. In addition to the methylation regulation, other epigenetic modifications have also been clarified later in ovarian cancer cells, such as the HDAC-induced histone deacetylation, which participates in regulating the expression of RGS10 in the ovarian cancer cells with chemoresistance [76]. Thus, one conclusion can be made that epigenetic mechanisms contribute to the regulation of *RGS* genes in different cancers.

### RGS protein in cancer

Cancer is marked by the uncontrolled growth of cells with up-regulated proliferation and down-regulated apoptosis [77], and is frequently accompanied by the enhanced capacity in cell migration, invasion and metastasis [78]. Those alterations are due to the changes in the activities of multiple cellular signaling pathways that can be regulated by certain molecules or proteins. Owing to the past achievements in establishment of the fundamental effects of GPCRs and heterotrimeric G proteins (the Gα and Gβγ subunits) on cancer occurrence and progression [79, 80], the potential role of RGS proteins in cancer has been recently unveiled. RGS proteins were first found to be associated with cancer in 2004, when it was discovered that a single nucleotide polymorphism (SNP) in the *RGS6* gene (rs2074647) was positively correlated with a decreased risk of bladder cancer, particularly in smokers [15].

Similar to the distribution pattern of GPCRs, RGS proteins are also widely expressed in many cells and tissues in humans [12, 81]. Previous studies have clarified a number of RGS proteins that linked to various cancers (summarized in Table 1) [74, 82–121]. Recent studies have further indicated the potential effects of RGS proteins on the initiation and progression of different cancers, where the RGS proteins act as tumor initiators or tumor suppressors, depending on the RGS protein that functions and the context of cancer [95, 119, 122–125]. For example, some RGS proteins including RGS4, RGS16, RGS2, RGS6 and RGS17 negatively regulate the progression of breast cancer, whereas RGS20 protein exerts the positive effects on potentiating the carcinogenesis of breast cancer [84, 105, 115, 126–128]. Moreover, RGS2 and RGS10 are found to inhibit cell proliferation in ovarian cancer [129, 130], whereas RGS19 gives rise to the opposite effect on ovarian cancer progression [131]. Intriguingly, the same RGS protein can also mediate cancer progression as the opposite role in different cancers. For example, in ovarian cancer cells RGS17 is found to be involved in the suppression of cancer cell growth and in the elevated responses to certain chemotherapeutic drugs [62]. However, RGS17 exerts positive effects on cancer cell growth in lung cancer, prostate cancer and hepatocellular carcinoma [72, 120, 132, 133]. In addition, despite the role of tumor suppressor in breast cancer [105], bladder cancer [104] and lung cancer [106], RGS6 is indicated to be a molecule that potentiates carcinogenesis, and particularly, dysregulation of RGS6 has been demonstrated to be positively associated with several cancers, including ovarian cancer [134] and pancreatic cancer [107]. In another example, down-regulation of RGS2 expression induces cell proliferation in ovarian cancer [129], whereas the opposite results occur in prostate cancer [86]. Although GAP activity is fundamentally regulated in RGS proteins-induced mediations, recent studies have provided with the evidence that the GAP activity from a simple G protein is not required in some RGS protein-mediated regulation effects. RGS4 functions in breast cancer cells through regulating its classical GAP activity, whereas RGS16 and RGS6 participate in the negative regulation of breast cancer cells in a GAP-independent manner [135]. The different mechanisms of RGS protein regulation implicate the possible methods for intervention and therapeutic treatment of related diseases. Take RGS4 protein discussed above as an example: in breast cancer cells, disruption of the interaction between RGS and G proteins will cause the selective inhibition of the GAP-dependent functions of RGS4, while targeting the expression of RGS4, RGS6 and RGS16 can give rise to the effects on both the GAP-dependent and GAP-independent regulations of these RGS proteins.

**Table 1:** Summary of the roles of different RGS proteins in cancers

Role of RGS	RGS Proteins	Cancers (References)
Tumor Initiator	RGS1	Gastric cancer [139], cervical cancer [140]
	RGS2	Prostate cancer [86]
	RGS3	Breast cancer [88], lung cancer [89], gastric cancer [65, 90], glioma [91]
	RGS4	Papillary thyroid cancer [92], lung cancer [94], glioma [95], osteosarcoma [97]
	RGS5	Squamous cell carcinoma [98], renal cell carcinoma [101]
	RGS6	Ovarian cancer [99, 134], pancreatic cancer [107]
	RGS7	Melanoma [108]
	RGS8	Prostate cancer [109]
	RGS11	Lung cancer [111]
	RGS16	Colorectal cancer [116], glioma [119]
	RGS17	Hepatocellular carcinoma [120], prostate cancer [72, 121, 138], lung cancer [132, 133, 138]
	RGS19	Ovarian cancer [131]
	RGS20	Breast cancer [127], bladder cancer [98, 136], penile cancer [141]
Tumor Suppressor	RGS1	Melanoma [82], multiple myeloma [83]
	RGS2	Breast cancer [84], colorectal cancer [85], bladder cancer [87], ovarian cancer [129]
	RGS4	Breast cancer [93], melanoma [96]
	RGS5	Ovarian cancer [99], liver cancer [100], lung cancer [102], perivascular soft tissue tumor [103]
	RGS6	Bladder cancer [104], breast cancer [105], lung cancer [106]
	RGS10	Ovarian caner [74, 130], colorectal carcinoma [110]
	RGS12	Oral squamous cell carcinoma [112], prostate cancer [113], osteosarcoma [114]
	RGS16	Breast cancer [115], pancreatic cancer [117], chondrosarcoma [118]
	RGS17	Breast cancer [128], ovarian cancer [62]

Aberrant expression of RGS proteins is observed in various cancers. It has been shown that in different cancers that alterations in functions of cancer cells to the expression patterns of specific RGS proteins are complicated, which can be deleterious but also can be wholesome. RGS2, RGS4 and RGS6 repress cell growth, whose expression is decreased in breast cancer cells, compared to the normal cells [84, 105, 126]. In contrast, RGS20 promotes cell growth and its expression is elevated in cancer cells, such as the bladder cancer [127, 136]. Similar phenomena can be also detected in prostate cancer cells, where RGS2 inhibits cell growth with the decreased RGS2 expression while RGS17 enhances cell growth with the increased RGS17 expression [72, 137, 138]. In addition, the expression of RGS12 is significantly reduced in oral squamous cell carcinoma (OSCC) tissues, and overexpression of RGS12 represses OSCC cell proliferation and migration through regulating the phosphorylation and SUMOylation of phosphatase and tension homolog (PTEN) [112]. Conversely, the expression of RGS1 is observed to be aberrantly up-regulated in gastric cancers and cervical cancers with poor prognosis in patients [139, 140]. In addition, the increased levels of RGS20 is correlated with tumor progression and unfavorable clinical outcome in penile cancer, where overexpression of RGS20 potentiates penile cancer progression through modulating the activity of PI3K/AKT signaling [141]. Similar phenomena can be observed in gastric cancer, where the expression levels of RGS3 is apparently increased and overexpression of RGS3 markedly promotes gastric cancer cell proliferation [65]. Thus, the alterations in RGS protein expression are strongly associated with carcinogenesis. On the other hand, emerging evidence has also indicated that the expression of RGS proteins is regulated by chemotherapeutic drugs [75, 142, 143], suggesting that the effects of RGS on cancer cell growth are accompanied by the progression and treatment of cancer. Table 2 summarizes multiple mechanisms underlying different RGS proteins in various cancers.

**Table 2:** Summary of mechanisms underlying RGS proteins in cancers

RGS proteins	Mechanism/Signaling pathway	Cancers (Reference)
RGS2	MCPIP1-dependent pathway	Breast cancer [84]
	Extracellular signal-regulated kinase 1/2 (ERK1/2)	Prostate cancer [137]
RGS4	Posttranslational regulation	Breast cancer [126]
RGS6	SNP (rs2074647)	Bladder cancer [15]
	p53 activation and DNMT1 downregulation	Bladder cancer [104]
	DNA-damage-induced apoptotic signaling	Breast cancer [105]
	Phosphatidylinositol 3-kinase signaling	Breast cancer [115]
	Mitochondrial-dependent pathway	Breast cancer [135]
	Interaction with SMAD4	Non-small cell lung cancers [106]
	A reactive oxygen species-dependent mechanism	- (MEFs)[142]
RGS10	AKT signaling	Ovarian cancer [75]
	Rheb-GTP and mTOR signaling	Ovarian cancer [130]
RGS12	Phosphorylation and SUMOylation of PTEN	Oral cancer [112]
RGS17	A target of miR-32	Breast cancer [128]
	A target of miR-199	Hepatocellular carcinoma [120]
	Cyclic AMP-PKA-CREB pathway	Lung and prostate cancer [138]
RGS19	Cell cycle control and AKT signaling	- (HEK293 cells) [131]
RGS20	NF-kappaB signaling	Bladder cancer [136]
	PI3K/AKT signaling	Penile cancer [141]

In summary, RGS proteins act as important mediators in cancers by regulating cancer cell functions and improper expression of RGS proteins are closely associated with cancer initiation and progression.

## Summary and perspectives

Despite that we have learned much from the findings of past work focused on several RGS proteins, overall efforts in targeting RGS proteins are woefully incomplete. RGS proteins have been shown to be involved in regulating kinds of cancers both in in vitro and in vivo studies, thus targeting different RGS proteins would be undoubtedly a promising method for cancer therapy. However, the structure and function of RGS proteins present a challenge. Since RGS proteins are mostly composed of protein–protein interaction domains, which themselves do not have an intrinsic biochemical activity that can be directly detected, researchers in the past 10 years have made great efforts in high-throughput screening and discovering potential inhibitors by focusing on small molecules targeting RGS-effector protein–protein interactions. Nevertheless, targeting protein–protein interactions with small molecules is a significant challenge, which has been considered intractable, thus a great deal more needs to be accomplished and it is exciting to look forward to what the next decade might bring in the study area of targeting RGS proteins. Considering the diverse function of GPCR in regulating cellular processes, having a better understanding of the mechanisms underlying RGS protein function might indirectly contribute to the development of therapeutics for the G protein signaling-associated diseases. On the other hand, given that the aberrant expression of RGS proteins is associated with cancer progression and that the expression of RGS proteins is ultimately determined by several different mechanisms including epigenetic, transcriptional and post-translational regulation, developing approaches with specific molecules targeting those mechanisms to restore the RGS protein expression to the desired levels would thereby provide with potential therapeutic targets for the intervention and treatment of those diseases.

## Data Availability

Not applicable.
